# Metabolomic and transcriptomic profiling reveals the alteration of energy metabolism in oyster larvae during initial shell formation and under experimental ocean acidification

**DOI:** 10.1038/s41598-020-62963-3

**Published:** 2020-04-09

**Authors:** Zhaoqun Liu, Yukun Zhang, Zhi Zhou, Yanan Zong, Yan Zheng, Chang Liu, Ning Kong, Qiang Gao, Lingling Wang, Linsheng Song

**Affiliations:** 1 Liaoning Key Laboratory of Marine Animal Immunology, Dalian Ocean University, Dalian, 116023 China; 2 Functional Laboratory of Marine Fisheries Science and Food Production Processes, Qingdao National Laboratory for Marine Science and Technology, Qingdao, 266235 China; 3 Liaoning Key Laboratory of Marine Animal Immunology and Disease Control, Dalian Ocean University, Dalian, 116023 China; 4 State Key Laboratory of Marine Resource Utilization in South China Sea, Hainan University, Haikou, Hainan China; 5 Dalian Key Laboratory of Marine Animal Immunology and Disease Control, Dalian Ocean University, Dalian, 116023 China

**Keywords:** Physiology, Climate sciences, Ecology, Ocean sciences

## Abstract

Marine bivalves secrete calcified shells to protect their soft bodies from predation and damages, which is of great importance for their survival, and for the safety of the coastal ecosystem. In recent years, larval shell formation of marine bivalves has been severely affected by ocean acidification (OA), and previous study indicated that OA might affect such process by disrupting endogenous energy metabolism. Developmental stages from trochophore to D-shape larvae are extremely important for initial shell formation in oyster since a calcified shell was formed to cover the chitin one. In the present study, metabolomic and transcriptomic approaches were employed to investigate the energy metabolism of oyster larvae during initial shell (prodissoconch I, PDI shell) formation and under experimental OA treatment. Totally 230 chemical compounds were identified from the present dataset, most of which were highly expressed in the “middle” stage (early D-shape larvae) which was critical for PDI shell formation since a calcified shell was formed to cover the chitin one. Several compounds such as glucose, glutarylcarnitine (C5), β-hydroxyisovaleroylcarnitine, 5-methylthioadenosine (MTA), myristoleate (14:1n5) and palmitoleate (16:1n7) were identified, which were involved in energy metabolic processes including amino acid oxidation, glycolysis, pentose phosphate pathway and fatty acid metabolism. In addition, mRNA expressions of genes related to protein metabolism, glycolysis, lipid degradation, calcium transport and organic matrix formation activities were significantly down-regulated upon experimental OA. These results collectively suggested that formation of the initial shell in oyster larvae required endogenous energy coming from amino acid oxidation, glycolysis, pentose phosphate pathway and fatty acid metabolism. These metabolic activities could be severely inhibited by experimental OA, which might alter the allocation of endogenous energy. Insufficient endogenous energy supply then suppressed the mobilization of calcium and resulted in a failure or delay in PDI shell formation.

## Introduction

The calcified shells are extremely important for marine bivalves living in the intertidal zone since the shells can protect them from tidal, predator and other harsh environmental factors^1,2^. Shell formation of marine bivalves happens as early as trochophore larvae, which relies on the energy from eggs^3,4^. According to previous study, total egg energy of the rock scallop *Crassadoma gigantea* expended during development was 11.8 kJ g^−1^ derived 46.7%, 9.8% and 43.5% from lipid, carbohydrate, and protein, respectively. It has been reported that 71% of the total energy obtained from parents is lost during metamorphosis in *Crassostrea virginica*^5^, and the lipid concentrations in *Mytilus edulis* embo larvae was also significantly lower than that in eggs^6^. Furthermore, much energy is also required for ion transportation to the site of mineralization, and to synthesize and secrete the organic polymers used in binding the minerals in the shell matrix^7^. These findings illustrated that the early shell formation of bivalve larvae requires sufficient endogenous energy sources such as glucose, lipids and protein. Lack of stored energy of imbalanced energy metabolism will result in a failure or delay in larval shell formation.

In recent years, ocean acidification has caused severe death of bivalve larvae by creating additional energetic costs^8^. To our best knowledge, OA imposes severely negative effects on bivalve larvae during the hours to days-long bottleneck when initial shell is formed during embryogenesis^9^. For example, bivalve larvae have to rely on endogenous energy during prodissoconch I shell formation stage^10,11^, and chronic acidification stress could severely inhibit such process^9^. OA was able to reduce the glycogen storage in juvenile oysters^12^, and Pearl oyster *Pinctada fucata* exhibited decrease in calcification rate upon acidifying treatment owing to lack of energy^13^. Also, the energy cost of calcification was empirically derived to be no more than 1.1 μJ (ng CaCO_3_)^−1^ in oyster larvae, and larval families showed variation in response to ocean acidification, with loss of shell size ranging from no effect to 28%, which indicated that resilience to OA might exist among genotypes^14,15^. These works evidenced that OA is likely to affect larval shell formation in marine bivalves by disrupting the balance of energy metabolism. In recent years, some progress has been made on revealing the metabolic bases of bivalve larvae under OA threat. However, much work is still needed to illustrate the precise metabolic pathways of lipids, carbohydrates and proteins during the formation of the initial shell (PDI shell).

Oysters are benthic species economically and ecologically important to the near-shore ecosystem. Successful shell formation of oyster larvae will contribute to the health of coastal ecosystem and the sustainable development of shellfish mariculture. Meanwhile, the developing larval shell of oyster has been regarded as an attractive model for elucidating the process of molluscan shell formation. In recent years, the initial shell formation of oyster larvae has been severely affected by OA, and the underlying mechanism is poorly understood. In the present study, we hypothesized that OA would affect energy metabolism in oyster larvae and impose negative consequences on the formation of the initial shell. To test this hypothesis we explored the impact of experimental acidification treatments on larvae of the Pacific oyster *Crassostrea gigas* with metabolic and transcriptomic approaches, hoping to (1) identify metabolites related to lipid/carbohydrate/protein metabolism during PDI shell formation; (2) illustrate the influences of experimental OA on energy metabolism; and (3) reveal the negative effects of OA on PDI shell formation.

## Results

### Metabolite summary and significantly altered biochemicals

**Figure 1:**
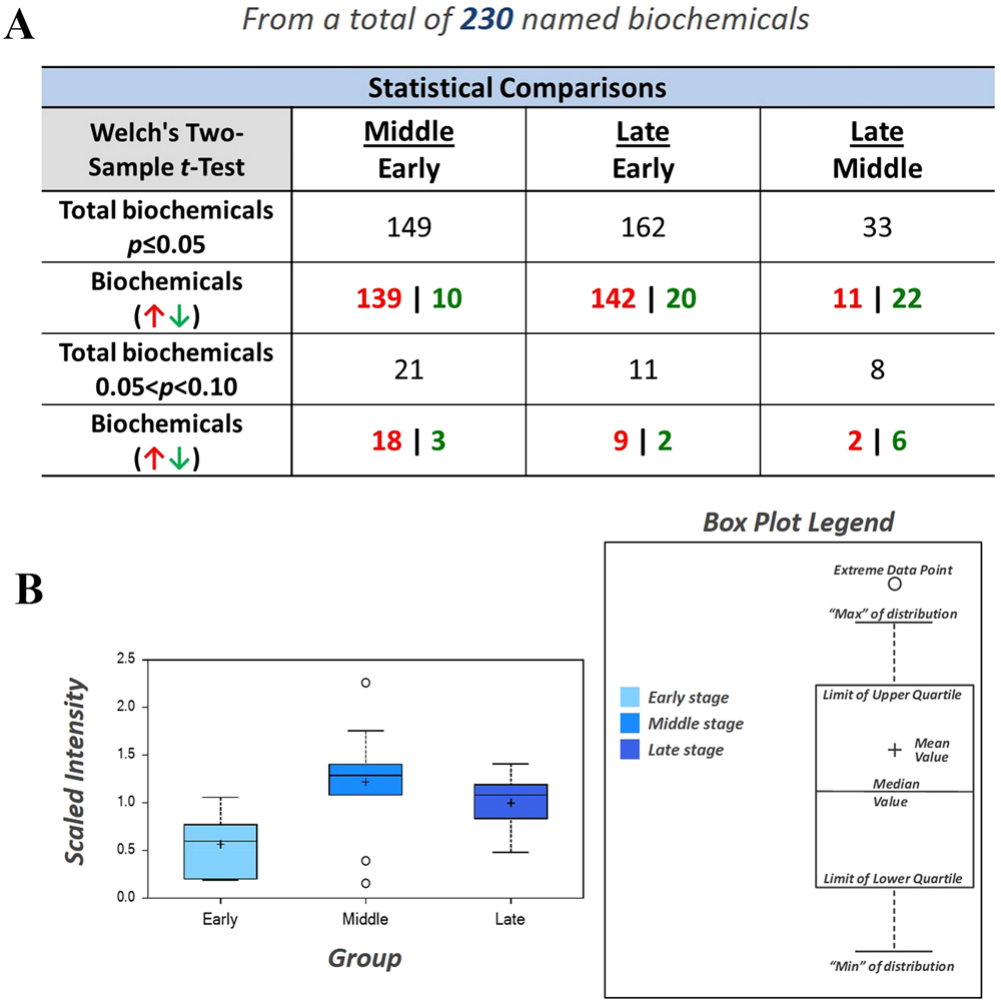
Total 230 chemical compounds identified by metabolomic approach. (**A**) The number of compounds identified from different developmental stages. (**B**) Data for each biochemical displayed as box plot. 149, 162 and 33 compounds were significantly expressed in the “early” (15 hpf), “middle” (17 hpf) and “late” stages, respectively.

Oyster larvae at three developmental stages (15 (“early”), 17 (“middle”) and 21 (“late”) hours post fertilization (hpf)) were collected for metabolomic analysis since they were the key stages for the formation of calcified shells in oyster larvae^2^. The present dataset comprised a total of 230 chemical compounds of known identity. 149, 162 and 33 compounds were identified from “early”, “middle” and “late” stages, respectively. A total of 149 chemical compounds were found to differentially expressed at the “early” stage, with 139 up-regulated ones and 10 down-regulated ones (Fig. 1A). The “middle” stage was critical for the formation of calcified shell in oyster larvae^2^, and most of the differentially expressed compounds were identified from this stage including 142 up-regulated ones and 20 down-regulated ones (Fig. 1A,B). At the “late” stage, the calcified shell was formed and relatively fewer compounds were identified, which consisted of 11 up-regulated ones and 22 down-regulated ones (Fig. 1A). The identified chemicals were shown in Table S1.

### Energy demand at key “middle” stage of larval development

**Figure 2:**
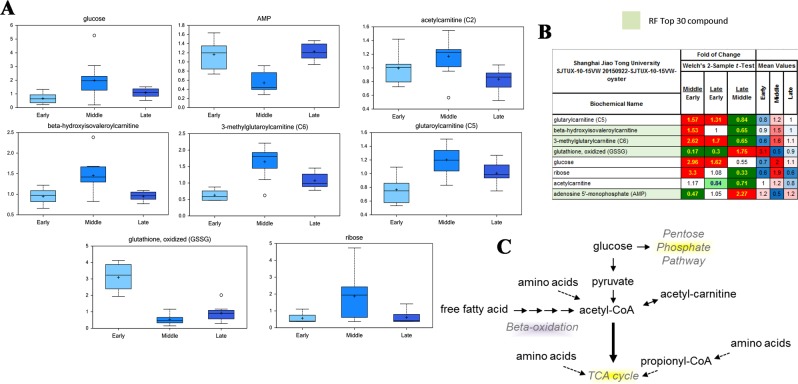
Energy demand at the key “middle” stage. (**A**) Expression of chemicals related to energy metabolism in different developmental stages. (**B**) Change fold of key chemicals related to energy metabolism. (**C**) The pentose phosphate pathway. Chemical compounds such as glucose, glutarylcarnitine (C5), β-hydroxyisovaleroylcarnitine, 3-methylglutarylcarnitine (C6) and acetylcarnitine (C2) and ribose were highly expressed in the “middle” stage, which was crucial for the formation of calcified shell.

Using Random forest and the Biochemical Importance Plot methods, several compounds related to energy metabolism were identified, most of which were highly expressed in the key “middle” stage at 17 hpf, during which the calcified shell was formed to cover the chitin one. Chemical compounds such as glucose, glutarylcarnitine (C5), β-hydroxyisovaleroylcarnitine, 3-methylglutarylcarnitine (C6) and acetylcarnitine (C2). On the other hand, ribose, a metabolite associated with the anabolic pentose phosphate pathway, was also significantly elevated during the “middle” developmental stage (Fig. 2).

### Identification of polyamines and nucleic acid metabolites

**Figure 3:**
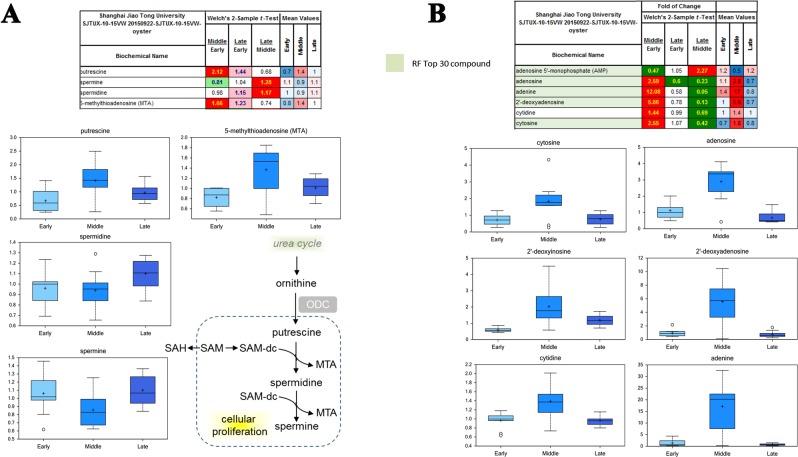
Metabolites of polyamines and nucleic acids metabolism. Many metabolites related to polyamines (**A**) and nucleic acid (**B**) metabolism were identified, including putrescine, 5-methylthioadenosine (MTA), adenosine, adenine, 2′-deoxyadenosine, cytidine and cytosine.

Many metabolites related to polyamines and nucleic acid metabolism were identified, including putrescine, 5-methylthioadenosine (MTA), adenosine, adenine, 2′-deoxyadenosine, cytidine and cytosine (Fig. 3). In particular, putrescine and MTA were involved in urea cycle and responsible for cellular proliferation process, while adenosine, adenine, 2′-deoxyadenosine, cytidine and cytosine were dramatically overexpressed in the “middle” stage and ranked the Top 30 chemicals identified in the present study.

### Higher fatty acid oxidation in the “middle” and “late” stages

**Figure 4:**
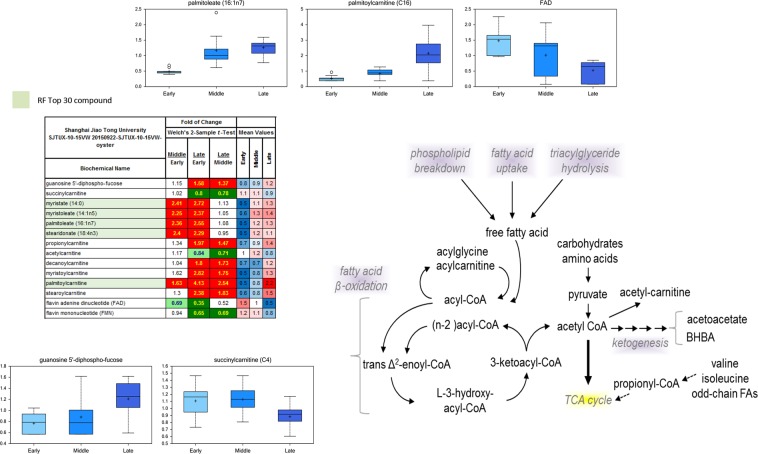
Metabolites of fatty acid oxidation in the “middle” and “late” stages. The expressions of succinylcarnitine, acetylcanitine and flavin mononucleotide (FMN) were significantly elevated in the “middle” stage, while propionylcarnitine, decanoylcarnitine, myristoylcarnitine, palmitoylcarnitine and stearoylcarnitine were highly expressed in the “late” stage. In addition, myristate (14:0), myristoleate (14:1n5), palmitoleate (16:1n7) and stearidonate (18:4n3) were highly expressed in both “middle” and “late” stages.

Free fatty acid metabolism was elevated in the “middle” and “late” stages comparing to the “early” stage (Fig. 4). The expressions of succinylcarnitine, acetylcanitine and flavin mononucleotide (FMN) were significantly elevated in the “middle” stage, while propionylcarnitine, decanoylcarnitine, myristoylcarnitine, palmitoylcarnitine and stearoylcarnitine were highly expressed in the “late” stage. Besides, myristate (14:0), myristoleate (14:1n5), palmitoleate (16:1n7) and stearidonate (18:4n3) were highly expressed in both “middle” and “late” stages.

### The differentially expressed genes related to energy metabolism in the key “middle” stage

**Figure 5:**
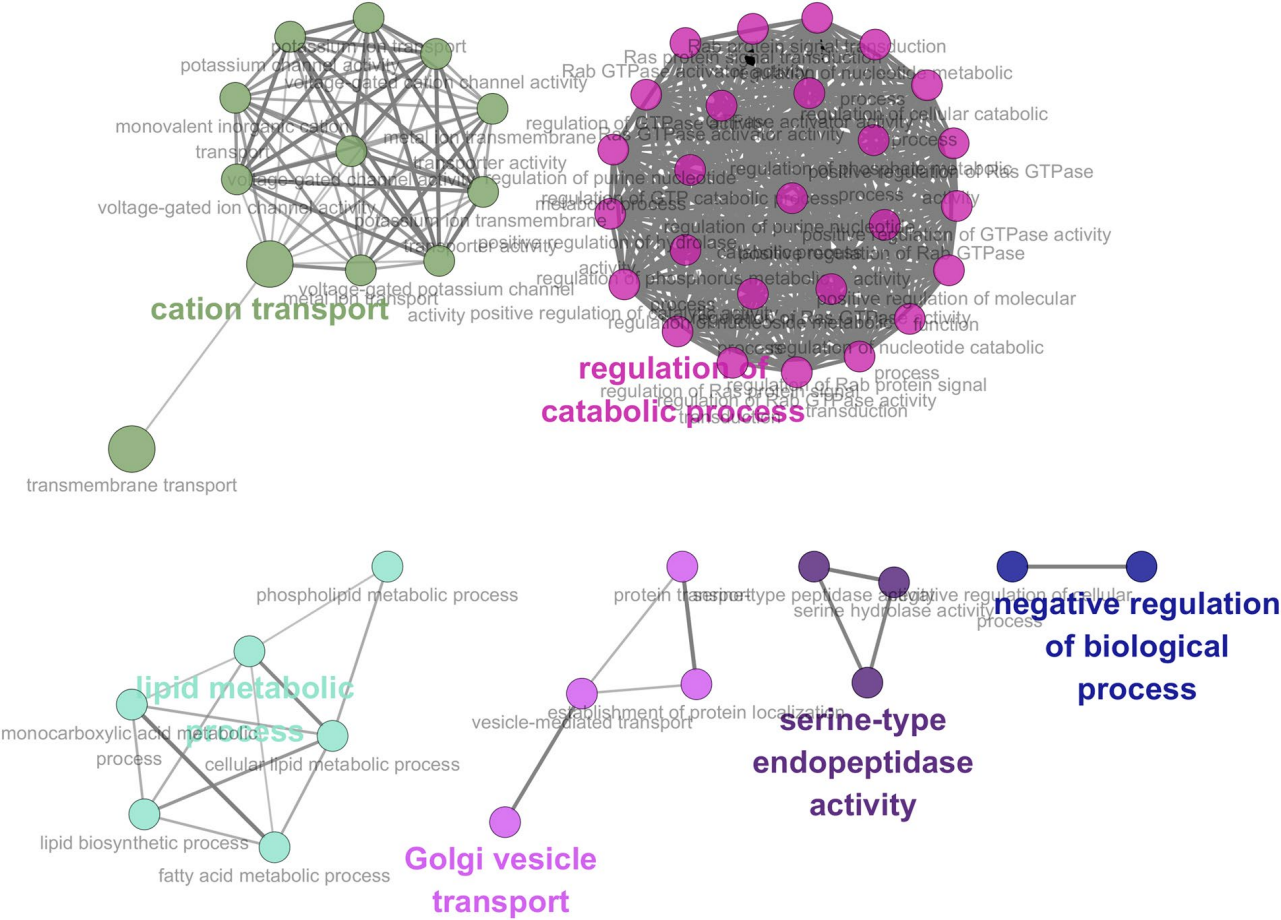
Overrepresented GO terms related to energy metabolism during calcified shell formation. Several GO terms related to energy metabolism were identified, including lipid metabolic process, cellular lipid metabolic process, lipid biosynthetic process, fatty acid metabolic process, monocarboxylic acid metabolic process, regulation of nucleoside metabolic process and regulation of purine nucleotide metabolic process. The overrepresented GO terms were displayed with Cytoscape 3.6.1 (http://cytoscape.org/).

For the significantly up-regulated genes identified from the “middle” stage, several GO terms related to energy metabolism and shell formation were identified, including lipid metabolic process (GO:0006629, FDR < 10.0E-3), cellular lipid metabolic process (GO:0044255, FDR < 19.0E-3), lipid biosynthetic process (GO:0008610, FDR < 96.0E-3), fatty acid metabolic process (GO:0006631, FDR < 69.0E-3), monocarboxylic acid metabolic process (GO:0032787, FDR < 120.0E-3), regulation of nucleoside metabolic process (GO:0009118, FDR < 140.0E-3) and regulation of purine nucleotide metabolic process (GO:1900542, FDR < 140.0E-3) (Fig. 5). Particularly, 10 and 7 genes characterized by overrepresented GO terms of lipid metabolic process and cellular lipid metabolic process were significantly overexpressed in the “middle” stage.

### Key processes affected by OA during larval shell formation

**Figure 6:**
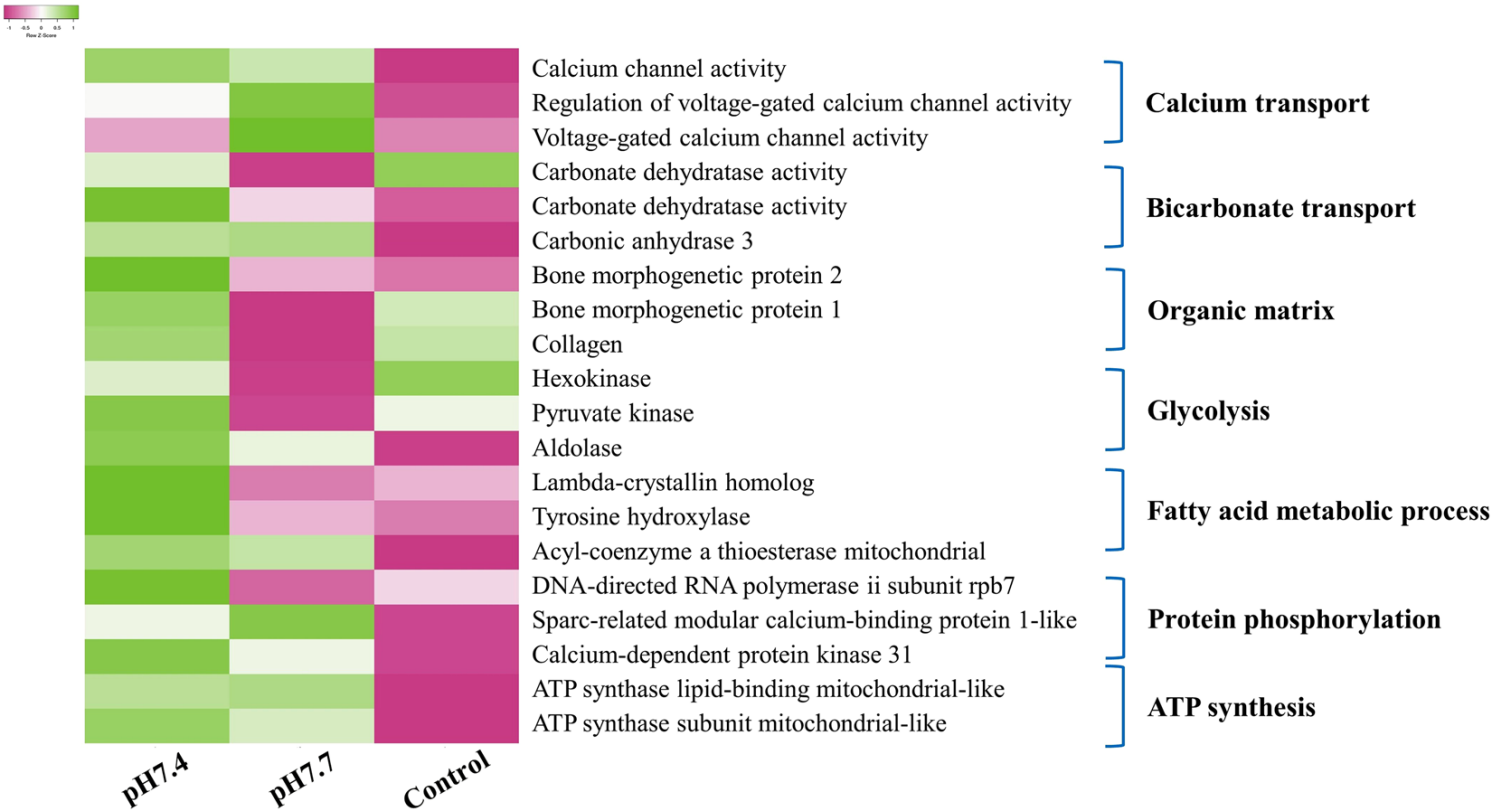
Key processes affected by experimental ocean acidification (OA) during larval shell formation. Experimental OA could suppress several key biological processes related to larval shell formation including included calcium transportation, bicarbonate transport, organic matrix, glycolysis, fatty acid metabolic process, protein phosphorylation, and ATP synthesis.

By analyzing the transcriptomic data of oyster larvae under experimental OA, expressions of genes related to key processes in larvae shell formation were significantly inhibited, which included calcium transportation (CGI_10000261, CGI_10011110 and CGI_10008043), bicarbonate transport (CGI_10004992, CGI_10027742 and CGI_10020143), organic matrix (CGI_10011133, CGI_10003192 and CGI_10002674), glycolysis (CGI_10007559, CGI_10003670 and CGI_10025556), fatty acid metabolic process (CGI_10008805, CGI_10021437 and CGI_10001878), protein phosphorylation (CGI_10002840, CGI_10015358 and CGI_10015881), and ATP synthesis (CGI_10004070, CGI_10006577 and CGI_10005007) (Fig. 6, Table 1).

**Table 1:** Key significantly down-regulated genes under experimental acidification treatment.

Treatments	GO ID	GO terms	Nr. genes
pH 7.8	GO:0006826	iron ion transport	3
	GO:0010466	negative regulation of peptidase activity	7
	GO:0030414	peptidase inhibitor activity	12
	GO:0030529	ribonucleoprotein complex	16
	GO:0042254	ribosome biogenesis	6
	GO:0043232	intracellular non-membrane-bounded organelle	26
	GO:0044281	small molecule metabolic process	36
	GO:1901564	organonitrogen compound metabolic process	36
pH 7.4	GO:0006412	translation	107
	GO:0008135	translation factor activity, nucleic acid binding	20
	GO:0008137	NADH dehydrogenase (ubiquinone) activity	5
	GO:0009059	macromolecule biosynthetic process	147
	GO:0010467	gene expression	149
	GO:0015986	ATP synthesis coupled proton transport	9
	GO:0016651	oxidoreductase activity, acting on NAD(P)H	8
	GO:0019538	protein metabolic process	162
	GO:0043170	macromolecule metabolic process	228
	GO:0044249	cellular biosynthetic process	175
	GO:0044260	cellular macromolecule metabolic process	206
	GO:0044267	cellular protein metabolic process	147
	GO:1901576	organic substance biosynthetic process	176

## Discussion

Marine bivalves such as oysters and scallops secrete calcified shells as a supporting frame for their soft bodies and for protection from predators^1,2^, which is thought to be one of the key factors that trigger the expansion of bivalves at the dawn of the Cambrian times^16^. The early shell formation of bivalve larvae requires sufficient endogenous energy sources such as glucose, lipids and protein. In recent years, larval shell formation of marine bivalves has been severely affected by OA, resulting in vast mortality worldwide. Since previous study demonstrated that the level of reserves was only slightly higher than that required for shell formation in bivalve larvae^10^, it was hypothesized that OA might influence larval shell formation by disrupting the energy metabolic process. Although some progress has been made on revealing the metabolic bases of bivalve larvae under OA threat, much work is still needed to illustrate the precise metabolic pathways of lipids, carbohydrates and proteins during the formation of initial shell (PDI shell). In the present study, metabolomic and transcriptomic approaches were employed to study the metabolic variations in oyster larvae during formation of calcified shell and upon experimental OA, hoping to identify metabolites related to lipid/carbohydrate/protein metabolism during PDI shell formation and to reveal the negative effects of OA on oyster larvae from a point of view of energy metabolism.

Oyster larvae at three developmental stages (15 hpf (“early”), 17 hpf (“middle”) and 21 hpf (“late”)) were collected for metabolomic analysis since they were the key stages for the formation of calcified shells in oyster larvae. Totally 230 chemical compounds were identified from the present dataset, most of which were highly expressed in the “middle” stage. Overexpression of these metabolites indicated that a high level of energy metabolism was demanded for the formation of calcified shell. Using Random Forest and the Biochemical Importance Plot methods, a vast array of differentially expressed compounds related to energy metabolism at the “middle” stage were identified, such as glucose, glutarylcarnitine (C5), β-hydroxyisovaleroylcarnitine, 3-methylglutarylcarnitine (C6) and acetylcarnitine (C2) and ribose (Fig. 2). β -hydroxyisovalerylcarnitine, 3-methylglutarylcarnitine, glutarylcarnitine and acetylcarnitine are key intermediates of protein phosphorylation and amino acid oxidation^17^, while putrescine, 5-methylthioadenosine (MTA) and spermine were involved in the urea cycle^18^. Amino acids, derived largely from protein in the diet or from degradation of intracellular proteins, are the final class of biomolecules whose oxidation makes a significant contribution to the generation of metabolic energy^19^. Meanwhile, amino acid catabolism results in waste ammonia, which needs a way to be excreted since they are toxic^20^. As for most aquatic organisms, they excrete ammonia by diluting it by water outside the organism or converting it into a less toxic substance such as urea or uric^21^. In the present study, metabolites of both amino acid oxidation pathway and urea cycle pathway were overexpressed at the early D-shape larvae (“middle”) stage, which was key period for the PDI shell formation since a calcified shell was formed to cover the chitin one^2^. Besides, experimental OA was able to significantly suppress the mRNA expression of genes including DNA-directed RNA polymerase II submit spb 7, sparc-related modular calcium-binding protein 1-like, calcium-dependent protein kinase 31, ATP synthase lipid-binding mitochondrial-like, and ATP synthase subunit mitochondrial-like, which were related to protein metabolism and ATP synthesis^22^. Besides, research in marine bivalves proved that the interaction of seawater acidification and elevated temperature led to further expression of amino acid metabolism^23^. Therefore, results in the present study suggested that protein metabolism by amino acid oxidation could be a critical source of endogenous energy for the formation of initial shell in oyster larvae. OA inhibited larval shell formation by suppressing amino acid metabolism and resulted in a lack of ATP synthesis, which might then cause a failure of delay in PDI shell formation.

Apart from amino acid oxidation, glycolysis and pentose phosphate pathway were also found to be significant energy sources for initial shell formation in oyster larvae^11,24^. Glucose and ribose were highly expressed in the “middle” stage. Glucose is the most important source of energy in all organisms. In the present study, metabolites related to glucose metabolism through glycolysis and pentose phosphate pathway were identified during initial shell formation, and transcriptomic analysis illustrated that the expression genes related to glycolysis, such as pyruvate kinase, hexokinase and aldolase, was obviously inhibited upon experimental OA. These results were inconsistent with previous reports that OA could up-regulate energy metabolism in both adult and larval marine bivalves. For example, it was said that when oyster *C*. *gigas* received an acute OA treatment, the alanine and ATP levels in mantle tissue decreased significantly whereas an increase in succinate levels was observed in gill tissue^25^. Thus, results from the present and previous studies suggested that glucose metabolism through glycolysis and pentose phosphate pathway should be another crucial source of energy supply for initial shell formation in oyster, which could be severely affected by OA and resulted in a failure or delay in larval shell formation.

Furthermore, fatty acids metabolism was found to be the third energy source for PDI shell formation in oyster larvae. On one hand, metabolites related to fatty acids metabolism including succinylcarnitine, acetylcanitine, flavin mononucleotide (FMN), myristate (14:0), myristoleate (14:1n5), palmitoleate (16:1n7) and stearidonate (18:4n3) were highly expressed in the “middle” stage. On the other hand, several GO terms related to fatty acids related were also identified from the transcriptomic data, including phospholipid metabolic process, fatty acid metabolic process, and cellular lipid metabolic process (Fig. 5). In addition, experimental OA could suppress mRNA expression of lambda-crystallin homolog, tyrosine hydroxylase and acyl-coenzyme a thioesterase mitochondrial, which were key molecules for fatty acid metabolism^26^. Fatty acids yield the most ATP on an energy per gram basis, when they are completely oxidized to CO_2_ and water by beta oxidation and the citric acid cycle^27^. The metabolites identified in the present study were responsible for the activation of fatty acid degradation and could induce an increase of ATP synthesis. However, the metabolism of lipids could be negatively regulated by OA. In the pearl oyster *P*. *fucata*, genes associated with the “fatty acid biosynthesis” pathways were significantly enriched under acidification treatment^13^. And, unigenes involved in “fatty acid metabolism”, but not “glycerol metabolism”, are differentially expressed upon pH 7.8 treatment in *P*. *fucata*^28^. Like proteins and glucose, balanced lipid metabolism was also critical for PDI shell formation in oyster larvae^24^. And, results in the present study indicated that OA might influence initial shell formation of oyster larvae by inhibiting the processes of fatty acid metabolism. Furthermore, activities related to “fatty acid metabolic process” was slightly inhibited upon moderate acidification treatment (pH 7.7) and severely inhibited upon severe acidification treatment (pH 7.4) comparing with normal group, suggesting that moderate acidification treatment could barely influence fatty acid metabolism, while such process could be dramatically suppressed under severe acidification treatment.

The above results evidenced that formation of the initial shell in oyster larvae required endogenous energy supplied by amino acid oxidation, glycolysis, pentose phosphate pathway and fatty acid metabolism. These metabolic activities were severely affected by experimental OA, resulting in a failure or delay in PDI shell formation. In order to reveal the link between energy metabolism and shell formation, the expression patterns of shell formation-related genes were further investigated. By analyzing the transcriptomic data of oyster larvae under experimental OA, expressions of genes related to key processes in larvae shell formation were significantly inhibited, which included calcium transportation, bicarbonate transport, and organic matrix. These results suggested that the mobilization of calcium in oyster larvae was significantly disrupted upon experimental OA treatment. According to our previous study, oyster carbonic anhydrases (CA) was able to modulate intracellular pH (pHi) of oyster haemocytes under CO_2_ exposure^29^. Meanwhile, it was found that elevated CO_2_ caused the decrease of intracellular Ca^2+^ in haemocytes. The inhibition of CA by acetazolamide and suppression of *Cg*CA gene via RNA interference increased the intracellular Ca^2+^ in haemocytes^30^. The above results suggested that initial shell formation in oyster larvae required endogenous energy supplied by amino acid oxidation, glycolysis, pentose phosphate pathway and fatty acid metabolism. Experimental OA affected such process by disrupting the balanced energy metabolism, which might alter the allocation of metabolic energy and further suppress the mobilization of calcium in oyster larvae. Moreover, activities related to “organic matrix” and “glycolysis” was activated upon moderate acidification treatment (pH 7.7) and inhibited upon severe acidification treatment (pH 7.4), which indicated that moderate acidification treatment could activate the stress response in oyster to sustain homeostasis upon acidifying environment, while the severe acidification treatment would significantly suppress normal physiological activities.

**Figure 7:**
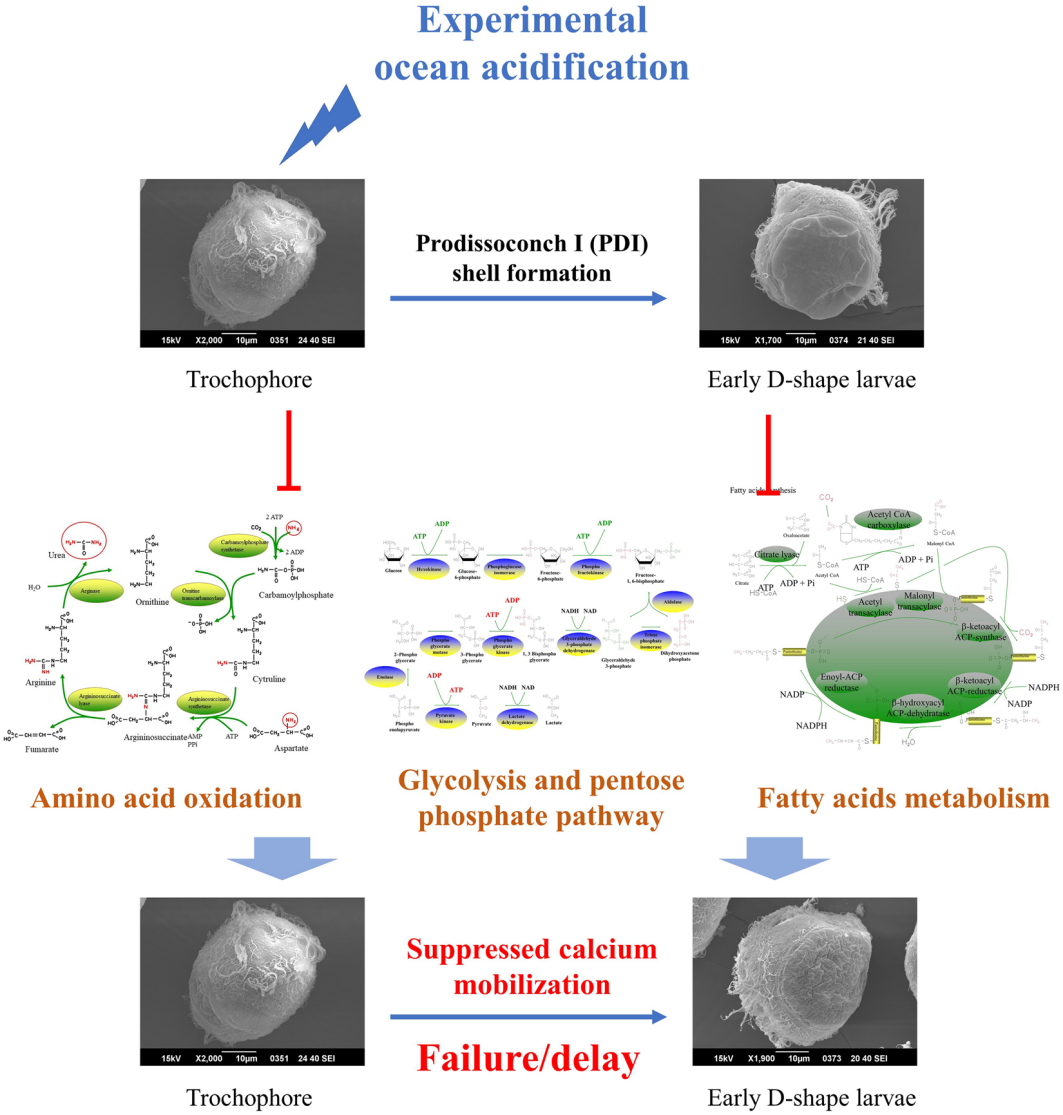
Schema illustrating how OA affected initial shell formation in oyster larvae. The formation of the initial shell in oyster larvae required endogenous energy coming from amino acid oxidation, glycolysis, pentose phosphate pathway and fatty acid metabolism. These metabolic activities could be severely inhibited by experimental OA, which might alter the allocation of metabolic energy. Insufficient endogenous energy supply then suppressed the mobilization of calcium and resulted in a failure or delay in PDI shell formation.

In conclusion, metabolomic and transcriptomic approaches were employed to investigate the energy metabolism of oyster larvae during PDI shell formation and under experimental OA treatment (Fig. 7). Results in the present study suggested that formation of the initial shell required endogenous energy coming from amino acid oxidation, glycolysis, pentose phosphate pathway and fatty acid metabolism. These metabolic activities could be severely inhibited by experimental OA, which might alter the allocation of metabolic energy. Insufficient endogenous energy supply then suppressed the mobilization of calcium and resulted in a failure or delay in PDI shell formation.

## Methods

### Oyster larvae sample collection

Sexually mature oyster *C*. *gigas* (about 2-year old, averaging 150 mm in shell length) were collected from a local farm in Dalian, Liaoning Province, China, and maintained in the aerated seawater at 20°C for 14 days before processing. The eggs and sperms were scraped from different adult oysters and mixed well to minimize individual variability. After fertilization, the developing embryos were cultured in filtered and aerated sea water at 20 °C. The breeding protocol was performed according to previous description^31^.

In the acidification treatment experiment, trochophores collected at 15 hours post fertilization (hpf) were equally divided into two groups, taking three replicates for each group. Larvae cultured in normal sea water (pH = 8.10 ± 0.05) was designated as the control group (Normal group), while those in the CO_2_ exposure groups (pH7.7 and pH7.4 groups) were bathed in acidified seawater (pH = 7.40 ± 0.05)^32,33^. The pH value of the CO_2_ exposure group was controlled using an acidometer (AiKB, Qingdao, China)^34^. Total alkalinity was determined by end-point titration of 25 mmol L^−1^ HCl on 50 mL samples. Carbonate parameters were calculated from the pH and total alkalinity.

The collection of larvae was performed according to Liu *et al*.^31^. Trochophore, early D-shape larvae and D-shape larvae were sampled at 15 hpf, 17 hpf, and 21 hpf, respectively. And, early D-shape larvae under experimental ocean acidification treatments were also collected. One microliter of Trizol (Invitrogen) was added to each tube containing larvae for RNA isolation, while larvae for metabolomic analysis were frozen directly in liquid nitrogen. Three replicates were conducted for RNA sequencing, while ten replicates were employed for each group in metabolomic analysis.

### Metabolite profiling

Trochophore, early D-shape larvae and D-shape larvae sampled at 15 hpf, 17 hpf, and 21 hpf respectively were sent to metabolomic sequencing to explore the metabolic patterns in oyster larvae during shell formation. The significant compounds were identified with liquid/gas chromatography coupled to mass spectrometry (LC/GC–MS) method. The informatics system consisted of four major components, the Laboratory Information Management System (LIMS), the data extraction and peak-identification software, data processing tools for QC and compound identification, and a collection of information interpretation and visualization tools for use by data analysts. The hardware and software foundations for these informatics components were the LAN backbone, and a database server running Oracle 10.2.0.1 Enterprise Edition. Raw data was extracted, peak-identified and QC processed using Metabolon’s hardware and software. The informatics system used in the present study was described by Luo *et al*.^35^. Random Forest method was used to analyze the metabolomic data^36,37^.

### Bioinformatical analysis of the transcriptomic data

Trochophore, early D-shape larvae and D-shape larvae sampled at 15 hpf, 17 hpf, and 21 hpf, as well as D-shape larvae under moderate (pH 7.7) and severe (pH 7.4) acidification treatment were sent to RNA sequencing. The transcriptomic data was analyzed to explore the metabolic patterns in oyster larvae during shell formation. The analyzing protocol was similar to previous description^38,39^. Rstudio and Cytoscape ClueGO software were adopted to perform Gene Ontology (GO) overrepresentation analysis of the identified significantly up- and down-expressed genes. The hypergeometric test with FDR value of 0.01 was used to do the GO enrichment, and the differentially expressed genes were selected as test set, while all identified genes were taken as the reference set. The significantly overrepresented GO terms were calculates from test set, and displayed as a network using BiNGO plug-in to Cytoscape (http://cytoscape.org/)^38^.

### Statistical analysis

Statistical analysis was performed and all data were given as Means ± S.D. Statistical significance was determined by two-tailed Student’s t-test, or by one-way analysis of variance (ANOVA) followed by S-N-K post hoc test for multiple comparisons. Statistically significant difference was designated at *p* < 0.05, indicating by asterisks.

## Supplementary information

Supplementary Information.
